# Supplementary material for: Rapid turnover of life-cycle-related genes in the brown algae
Source: Genome Biol. 2019 Feb 14;20:35. doi: 10.1186/s13059-019-1630-6 (PMC6374913; doi:10.1186/s13059-019-1630-6)
Supplement: Supplementary file 3 — Protein alignments, corresponding Gblocks html file and CDS sequences for the lineage conserved gametophyte-biased genes. (ZIP 2109 kb) [file 13059_2019_1630_MOESM3_ESM.zip › Con_GA_alignments/Ec-02_001670.aa_ali.fasta-gb.htm]

Ec-02\_001670.aa.fasta.trans.aa\_ali.fasta


## Gblocks 0.91b Results

Processed file: **Ec-02\_001670.aa.fasta.trans.aa\_ali.fasta**  
Number of sequences: **2**  
Alignment assumed to be: **Protein**  
New number of positions: **642** (selected positions are underlined in blue)

```
                         10        20        30        40        50        60
                 =========+=========+=========+=========+=========+=========+
Ec-02_001670     MTSASSEKGNAAMSASVGEEGPWGHSDIFREPTDPEMIAYLGRKRESLQNEVVNVAQGMS
MSTRG.18212.1    ------------------------------------------------------------
                                                                             


                         70        80        90       100       110       120
                 =========+=========+=========+=========+=========+=========+
Ec-02_001670     IGPALYLWTHRLTGADVVSVVTTTRPSTSASTTTTHKTKYSAGGGLPVGSVDGGRGVLRR
MSTRG.18212.1    ------------------------------------------------------------
                                                                             


                        130       140       150       160       170       180
                 =========+=========+=========+=========+=========+=========+
Ec-02_001670     GPPFDGSFGRLEGGGKMPLQRRASAKWTILPKNKEASGEGGFMAVKGGKTDAELRRLRNE
MSTRG.18212.1    --------------------------------------------XKGGRAKAELKRLQRE
                                                              ###############


                        190       200       210       220       230       240
                 =========+=========+=========+=========+=========+=========+
Ec-02_001670     KNLRAPLHLPLFTSPLEVDPTGSGKVHDRMSECLMAERERFMELLGKERQERVHLEAFAC
MSTRG.18212.1    KRLRAPLQLPVFTSPLEAGPTGAGKAHDRMSECLMAERDCFMDLLGKERQERLHLEAFAC
                 ############################################################


                        250       260       270       280       290       300
                 =========+=========+=========+=========+=========+=========+
Ec-02_001670     SLVQAAFRGYLLRKRWAEVDARQRVRLRVRKCLRDFLKSRGAPFALGALEKQQKEDRLRQ
MSTRG.18212.1    SLVQAAYRGYLLRKRWAEVEARQRVRLRVRKSLREFLKSRGAPFALGALEKQQKEDNVRR
                 ############################################################


                        310       320       330       340       350       360
                 =========+=========+=========+=========+=========+=========+
Ec-02_001670     AAACTIQASFRCFLARQTLGKRRLASFLLRRRRAARTLQCWGRYLFASRRLR--------
MSTRG.18212.1    AAASTIQGSFRSFLARQTLGKRRMASFLLRRRRAARTLQCWARYKFSCRRLRYTRMRAQA
                 ####################################################        


                        370       380       390       400       410       420
                 =========+=========+=========+=========+=========+=========+
Ec-02_001670     ---------------------VTGHRRRVVHSIAASMIQQQWRLRK----IQEVTKSRLF
MSTRG.18212.1    ASRSRSAMMIQRQYRRHLAARITGQRRRVVHSIAASMIQGQWRLRKTLEGIREVVKSRLF
                                       ########################    ##########


                        430       440       450       460       470       480
                 =========+=========+=========+=========+=========+=========+
Ec-02_001670     RSAAAIQRIVRGYVAWKKVGRQLTRVRGLRKTRAVYTLQKFIRRAQARQRVRRLQRRRRR
MSTRG.18212.1    RSSAAIQRIARGYVARQKVGRQLTRVRALTRTRAVYLLQKFIRRAQARRRVNRLRERRRR
                 ############################################################


                        490       500       510       520       530       540
                 =========+=========+=========+=========+=========+=========+
Ec-02_001670     GTRARAALHVQRVCRGFVGRRRAAARKKATTLDIWRAAREGNASRVDALYYGQGLGGVIF
MSTRG.18212.1    GARARAALHVQRTCRGFVGRRRAAARKKSARLDIWRAAREGDASRVDALYYGQGLGGVIF
                 ############################################################


                        550       560       570       580       590       600
                 =========+=========+=========+=========+=========+=========+
Ec-02_001670     DCLNTKDPEGNSLLAKVASGVGPIALIRVVLKWGADVNSVNIRGESALQLAMSRRNVEVV
MSTRG.18212.1    DCLNNRDAEGNSLLAKVAGGAGPVSLVRVVLKWGADVNSANNRGETALRLAMSKRNVEVI
                 ############################################################


                        610       620       630       640       650       660
                 =========+=========+=========+=========+=========+=========+
Ec-02_001670     DLLVNHPNIRLNQDQPGKSILHEAAALGLHHLSAQLVSKGLDVNGVDPQNGWTPVHYACH
MSTRG.18212.1    DLLVNNPAIRLNAGEPGKSFLHEAAALGLHHLTAQLVSKGLDVNGVDPENGWTPVHYACK
                 ############################################################


                        670       680       690       700       710       720
                 =========+=========+=========+=========+=========+=========+
Ec-02_001670     NGRDELARLLTSRSGTMVNVGGAGGVTPMHLAAAGGWEKCATNLLENNASPVVKNNKGQV
MSTRG.18212.1    NGRDELARLLTSRSDTMVDLAGVGGATPLHLAASGGYAKCVTNLLENNASPVVKNNKGQV
                 ############################################################


                        730       740       750       760       770       780
                 =========+=========+=========+=========+=========+=========+
Ec-02_001670     PAVVAMLHGHASTGKKLLQAFSAAETASKRSRTIDAFAAVAMASGVTHAVEPVVDADKDE
MSTRG.18212.1    PAVVAMLHGHAPIAKNLLQAFAAAKTAPKRSHAMATIAGVALAAGVSHPSDPAPSADADG
                 ############################################################


                        790       800       810       820       830       840
                 =========+=========+=========+=========+=========+=========+
Ec-02_001670     NRSGGRQGSEKPGAGGGGGAGDGLVGLEGITTPEVASADVAEPAVVGEGGSDHNTPDDAG
MSTRG.18212.1    NP--GRPPDSSGGGDGGGGSGGGGGGPAGRRIAKLAATALAADAATAGGPAGGASPAPAN
                 #   ########################################################


                        850       860       870       880       890       900
                 =========+=========+=========+=========+=========+=========+
Ec-02_001670     SARGDRGLGAADGGASELQGGRPHSATTVGEKAGSTGSDASVDGGGRGGDAVSVQSNASS
MSTRG.18212.1    GSPGL-------------------------------------------------------
                 ####                                                        


                        910       920       930       940       950       960
                 =========+=========+=========+=========+=========+=========+
Ec-02_001670     RASASLEALSRRASKAGVAMDLNVIMLEDLVPRHWDDDDIRCAVALAERGEAWCLASLFA
MSTRG.18212.1    ------------------------------------------------------------
                                                                             


                        970       980       990      1000      1010      1020
                 =========+=========+=========+=========+=========+=========+
Ec-02_001670     LGLDPDCQHPDSGDTIGIAAARGGDRETLEVCLETGVSFATKNRRGRSALHAAVVRDDLE
MSTRG.18212.1    ------------------------------------------------------------
                                                                             


                       1030      1040      1050      1060      1070      1080
                 =========+=========+=========+=========+=========+=========+
Ec-02_001670     VAALLLANPGLSGIEVDDLCDPDEDGRTPLHEMAARGEAPTVLLLATEADSEWTTGGSGG
MSTRG.18212.1    ------------------------------------------------------------
                                                                             


                       1090      1100      1110      1120      1130      1140
                 =========+=========+=========+=========+=========+=========+
Ec-02_001670     AATGSGNASLDTRSEDASSAPGAGGGGGDDSSSGDDLSSSCVTAASSAVVDDQAGGVGGE
MSTRG.18212.1    ------------------------------------------------------------
                                                                             


                       1150      1160      1170      1180      1190      1200
                 =========+=========+=========+=========+=========+=========+
Ec-02_001670     NGARMGSGSSIPVSGGDTGVADGNTGDGAKRMSDIAAVASAAAAPDTPATSSVRSPLPLS
MSTRG.18212.1    ------------------------------------------------------------
                                                                             


                       1210      1220      1230      1240      1250      1260
                 =========+=========+=========+=========+=========+=========+
Ec-02_001670     AFVAPSLADTEELRDLRHHHHDVTNAAGKAERPEGVAMDTGRAGSTSTSTNVNSRSSLAR
MSTRG.18212.1    ------------------------------------------------------------
                                                                             


                       1270      1280      1290      1300      1310      1320
                 =========+=========+=========+=========+=========+=========+
Ec-02_001670     KASSSGDTRDTARSGGNHSDDGGDDHDDSGGREVRSEGEEGDDPSGWGGLLEVDVRCSGS
MSTRG.18212.1    ------------------------------------------------------------
                                                                             


                       1330      1340      1350      1360      1370      1380
                 =========+=========+=========+=========+=========+=========+
Ec-02_001670     GNCPLHEAAASGKVGAVLSLLDLGADVSVANGCGDTALHVAVGPNAPRDPMDEGHSRGST
MSTRG.18212.1    ------------------------------------------------------------
                                                                             


                       1390      1400      1410      1420      1430      1440
                 =========+=========+=========+=========+=========+=========+
Ec-02_001670     LASTSLSTLSTTSSPMVVGTTTATTTFSGDRRASGEAQDAMALCDDEGRGPTTQVVTALL
MSTRG.18212.1    ------------------------------------------------------------
                                                                             


                       1450      1460      1470      1480      1490      1500
                 =========+=========+=========+=========+=========+=========+
Ec-02_001670     RRGADPSKKNRLGQTAAMCAVLAGRAGALREIRKAVLLGHSSSKGKEQEELSDLGHMAIR
MSTRG.18212.1    ------------------------------------------------------------
                                                                             


                       1510      1520      1530      1540      1550      1560
                 =========+=========+=========+=========+=========+=========+
Ec-02_001670     EGHVGCLRVLTESCSEQEVLAWTDEEGVPALALAVARGQRECIDILLKGVSPTTSCGGDG
MSTRG.18212.1    ------------------------------------------------------------
                                                                             


                       1570      1580      1590      1600      1610      1620
                 =========+=========+=========+=========+=========+=========+
Ec-02_001670     GGGGGNTVLHVTAMARGGVPVLKRLMSPKGGGEPFDLGWLEYRNGLGDTVLMAALRAGRH
MSTRG.18212.1    -GG---------------------------------------------------------
                                                                             


                       1630      1640      1650      1660      1670      1680
                 =========+=========+=========+=========+=========+=========+
Ec-02_001670     SAALLLLQALAVPTAAAHTFRYAWVIAAALRLAELCPCPSCVLGRTADERARLCSKIAPK
MSTRG.18212.1    ------------------------------------------------------------
                                                                             


                       1690      1700      1710      1720      1730      1740
                 =========+=========+=========+=========+=========+=========+
Ec-02_001670     PTRPGEQSQGSHANPLSSAGASAAAAHRRLTRANSTASKAATTASDGALPAFVFPGSNPR
MSTRG.18212.1    ------------------------------------------------------------
                                                                             


                       1750      1760      1770      1780      1790      1800
                 =========+=========+=========+=========+=========+=========+
Ec-02_001670     RLRQLPRQRPGVAAAAAAVGRHLGRRRWRRKAKEACSSPLGGAVQGKGGRSAADGPMDVG
MSTRG.18212.1    ------------------VGV---------------------------------------
                                                                             


                       1810      1820      1830      1840
                 =========+=========+=========+=========+
Ec-02_001670     GPAGWKERRATTTALAAAAAAVAKSKRKPSPNAGVGRPRR
MSTRG.18212.1    ---------------------------------------H
```

```
Parameters used
Minimum Number Of Sequences For A Conserved Position: 2
Minimum Number Of Sequences For A Flanking Position: 2
Maximum Number Of Contiguous Nonconserved Positions: 8
Minimum Length Of A Block: 10
Allowed Gap Positions: None
Use Similarity Matrices: Yes
```

```
Flank positions of the 4 selected block(s)
Flanks: [166  352]  [383  406]  [411  781]  [785  844]  

New number of positions in Ec-02_001670.aa.fasta.trans.aa_ali.fasta-gb:  642  (34% of the original 1840 positions)
```
